# Synthesis of Nitrogen-Doped Lignin/DES Carbon Quantum Dots as a Fluorescent Probe for the Detection of Fe^3+^ Ions

**DOI:** 10.3390/polym10111282

**Published:** 2018-11-17

**Authors:** Xueqin Jiang, Yixin Shi, Xin Liu, Meng Wang, Pingping Song, Feng Xu, Xueming Zhang

**Affiliations:** Beijing Key Laboratory of Lignocellulosic Chemistry, Beijing Forestry University, Beijing 100083, China; xqjiang@bjfu.edu.cn (X.J.); shiyixinchn@bjfu.edu.cn (Y.S.); xin_liu@bjfu.edu.cn (X.L.); mwang@bjfu.edu.cn (M.W.); ppsong@bjfu.edu.cn (P.S.); xfx315@bjfu.edu.cn (F.X.)

**Keywords:** carbon quantum dots, lignin, deep eutectic solvent, fluorescent, iron ions

## Abstract

Carbon quantum dots (CQDs) as a rising star of carbon nanomaterials have extensive applications due to their excellent characteristics. In this work, we introduce a simple and green method to prepare nitrogen-doped lignin carbon quantum dots (N-L-CQDs) by using alkali lignin carbon sources and deep eutectic solvent (DES) as solution and nitrogen source. The physiochemical characterization results suggested that N-L-CQDs with diameters ranging from 4 to 12 nm were successfully synthesized. The optical properties data indicated that the as-prepared N-L-CQDs with a quantum yield of 7.95% exhibited excellent optoelectronic properties, excitation-dependent and pH stability. After that, we have investigated the N-L-CQDs used as fluorescent probes to detect iron ions, which suggested that the as-prepared N-L-CQDs exhibited excellent sensitivity and selectivity for Fe^3+^ with a detection limit of 0.44 μM. Besides, cytotoxicity of N-L-CQDs was also evaluated by MTT assay. These results demonstrated that the as-prepared N-L-CQDs with excellent properties have potential applications in environment and biomedicine.

## 1. Introduction

Carbon quanta dots (CQDs), can also be called organic carbon quantum dots, and are a new type of carbon nanomaterials. Comparing with traditional semiconductors quantum dots, they demonstrate some predominance in excellent optoelectronic properties, low toxicity, good biocompatibility, uncomplicated preparation method, photochemical stability, good dissolution, and low cost of preparation [1,2]. Therefore, CQDs have a wide range of applications, including fluorescence sensors [3,4], photocatalysis [5], and biosensing [6]. Besides, they can also substitute organic dyes for cell imaging [7].

CQDs have attracted increasing attentions for their excellent performance and extensive applications in recent years. Some groups have studied different synthetic methods of CQDs as well as the distinct precursor of CQDs. The method for preparing CQDs with fluorescent properties can be summarized into two kinds of approaches: “top-down” [8] and “bottom-top” [9]. “Top-down” methods refer to the formation of CQDs directly from larger size carbon-containing precursors, including arc discharge [10], laser ablation [8,11], and electrochemical methods [12,13]. The “bottom-up” approaches of synthesizing CQDs from small molecules precursors by methods like combustion preparation [14], hydrothermal treatment [15,16], and ultrasonic [17] and microwave routes [18,19]. However, most methods of synthesis CQDs demand special instruments, expensive or toxic starting materials, harsh synthesis conditions, and complex steps [20,21,22]. Therefore, developing greener and simple synthetic approaches of CQDs with desired properties are urgently needed.

One of the characteristics of green synthesis is the use of renewable and green bio-precursors as starting materials. A variety of renewable precursors have been selected as starting materials for the synthesis of CQDs in recent years, like banana juice [15], milk [16], honey [23], etc. In order to make full use of biowaste and reduce waste of resources, some people have researched converting biowaste into fluorescent bio-asset. The biowastes have been used as precursors for the preparation of CQDs such as plant leaf [24], waste of wine fermentation [25], and waste paper [26]. However, there is still a lot of biowaste that has not been fully used. Remarkably, technical lignin is produced in large quantities per year, including lignosulfonate and alkali lignin, whereas the efficient utilization of lignin presents an ongoing challenge [27,28]. Therefore, some researchers have investigated the preparation of quantum dots from lignin biopolymers. Xu et al. have successfully prepared CQDs derived from ligninsulfonate/graphene quantum dots composites as a fluorescent sensing for detection of Fe^3+^ ions [29]. Moreover, the CQDs from pyrolyzed alkaline lignin were prepared and the CQDs/TiO_2_ system can be used as an effective photocatalyst [30]. Recently, Ding et al. synthesized graphene quantum dots (GQDs) from alkaline lignin, the prepared GQDs with the properties of excellent photoluminescence can be used for multicolored bioimaging [31]. Therefore, the quantum dots synthesized from lignin not only showed excellent optical properties but also possessed widespread applications. Besides, lignin with the properties of nontoxic, renewable, inexpensive, and biodegradable can be considered as the most attractive sustainable carbon to synthesize CQDs [32,33].

Unfortunately, the surface defects of CQDs affect their photoluminescent intensity (PL), which ultimately leads to fluorescence quantum yield (QY) of CQDs as low as 1 to 2% or even less [8,34]. Recent studies have showed that doped heteroatoms (such as N, S, Si, etc.) can enhance the QY of CQDs [35,36]. Additionally, some experimental and theoretical results have confirmed that heteroatoms doped are an effective method for improving electrochemical and optical properties of CQDs [37,38], especially nitrogen-doped (N-doped); the most widely used doping methods [39]. In comparison with complicated synthesis of nitrogen-doped CQDs [22], direct dissolution of lignin with solvents containing nitrogen elements would be attractive and meaningful. As a newly emerged solvent, deep eutectic solvents (DESs) have properties of nontoxic, biodegradable, inexpensive, recyclable, and low vapor emission properties [40]. In general, DESs consist of two or more compounds that are capable of self-association through hydrogen bond interactions, forming a eutectic mixture with a final melting point much lower than the individual precursor components [41]. DESs can be obtained by mixing hydrogen bond acceptors (HBA, such as quaternary ammonium) with hydrogen bond donors (HBD, for instances, acids, alcohols, amines, etc.) [42]. Interestingly, DESs, possessing superior dissolving ability on lignin, have been used in the lignocellulose pretreatment stages [40,43]. Therefore, DES with ammonium acceptors can be used for dissolution of lignin to synthesize nitrogen-doped CQDs.

**Scheme 1:**
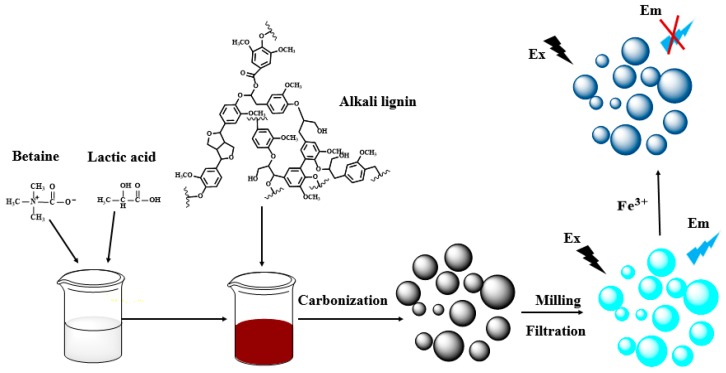
Schematic representation of synthesis of N-L-CQDs and illustration of the fluorescence quenching.

Herein, we present a green and simple method for synthesizing N-L-CQDs using deep eutectic solvent (DES) as the solvent for the dissolution of alkali lignin and carbonization treatment (Scheme 1). In the present study, alkali lignin was used as the main carbon sources and DES composed of betaine and lactic acid acted as both carbon and nitrogen sources. The as-prepared nitrogen-doped lignin carbon quantum dots (N-L-CQDs) displayed excellent fluorescence, low toxicity, and photochemical stability. Besides, the N-L-CQDs also exhibited superior sensitive and selective for Fe^3+^. Moreover, the substantial fluorescence quenching of the N-L-CQDs solution was observed with the addition of Fe^3+^ (Scheme 1). Therefore, the synthesis of N-L-CQDs s as fluorescent sensors offer a simple, sensitive, selective, easy to handle, and low-cost approach for assaying metal ions in biological and environmental aspects.

## 2. Materials and Methods

### 2.1. Materials

Alkali lignin was purchased from Shandong Longlive Bio-Technology Co., Ltd. (Shandong, China). All chemicals, including betaine (Shanghai Yuanye Biotechnology Co., Ltd., Shanghai, China), lactic acid (Shanghai Macklin Biochemical Co., Ltd., Shanghai, China), hydrochloric acid (Beijng Chemical Works, Beijing, China), sodium hydroxide (Beijing Chemical Works), and quinine sulfate dehydrate (Shanghai Macklin Biochemical Co., Ltd.) were used without further purification.

### 2.2. Synthesis of DES and Dissolution of Alkali Lignin

DES was prepared by mixing 8 g of betaine and 21.7 g of lactic acid in a 250 mL beaker and heating at 60 °C under magnetic stirring for about 10 min to obtain a clear and colorless liquid. 11.8 g of Alkali lignin was gradually added to DES and stirred for 12 h at 60 °C. The mixture eventually formed a viscous and deep brown solution.

### 2.3. Synthesis of Nitrogen-Doped Lignin Carbon Quantum Dots (N-L-CQDs)

N-L-CQDs was prepared via carbonization of the viscous solution at 300 °C for 30 min at a heating rate of 5 °C/min in a muffle furnace. When the temperature of muffle furnace dropped to 30 °C, the dark brown products were mechanically ball milled into powders. Then the powders were washed by distilled water and the supernatant contained the N-L-CQDs was obtained by vacuum filtration. The resulting bright yellow solution was evaporated in a rotary evaporator at 55 °C and dried under vacuum at 60 °C to obtain N-L-CQDs fine powders. Then the powders were dispersed in distilled water as N-L-CQDs solution (0.1 mg/mL) for further characterization and use.

### 2.4. Characterizations

The atomic force microscopy (AFM) image was recorded by a Bruker MultiMode 8 AFM (Bruker Corporation, Madison, WI, USA). Transmission electron microscopy (TEM) and the high-resolution transmission electron microscopy (HR-TEM) photographs were obtained on a Tecnai G2 F30 S-TWIN instrument (FEI Company, Hillsboro, OR, USA) with an accelerating voltage of 300 kV. X-ray diffraction (XRD) pattern of sample was performed with a BrukerAXS D8 (Bruker Corporation, Karlsruhe, Germany) with Cu radiation (40 kV, 40 mA, λ = 0.154 nm). Fourier-transform infrared spectrometry (FTIR) was conducted on a Nicolet iN10 FTIR using KBr pellets. The chemical states of the sample were analyzed by X-ray photoelectron spectroscopy (XPS) experiment was showed with an ESCALAB 250Xi photoelectron spectrometer using Al Kα source. The electronic absorption spectra in the ultraviolet-visible (UV–Vis) region were measured on a UV2310II spectrometer. The photoluminescence (PL) spectra were carried out on a Hitachi F-7000 spectrofluorometer with a Xe lamp as an excitation source. The PL decay curve were obtained by a steady state and transient state fluorescence spectrometer (FLS980, Edinburgh instruments, Edinburgh, UK) excited with a nanosecond laser at 300 nm.

#### 2.4.1. The Measurement of Quantum Yield of N-L-CQDs

The quantum yield of N-L-CQDs was measured by using quinine sulfate in 0.1 M H_2_SO_4_ solution as the standard (quantum yield is 0.54). The UV–Vis absorption peaks of the as-prepared N-L-CQDs solution and the quinine sulfate solution were kept below 0.1. The fluorescence peak areas were obtained by measuring PL spectra. The UV–Vis absorption and PL spectra were recorded at a maximum excitation wavelength of 300 nm. Then the quantum yield was calculated according to the following formula.

$$Q=Q_{s}\frac{I}{I_{s}}\times \frac{A_{s}}{A}\times \frac{\eta^{2}}{\eta_{s}^{2}}\times 100\%$$

where *Q* is the fluorescence quantum yield, *I* is the integral area of fluorescence peak, *A* is the absorbance, η refers to the refractive index of the respective solvents, and *s* stands for the reference of known quantum yield.

#### 2.4.2. Effect of pH, Temperature, and Time on the Fluorescence Sensing of N-L-CQDs Aqueous Solutions

The prepared N-L-CQDs solutions were adjusted to different pH values by using 1 M sodium hydroxide (NaOH) and 1 M hydrochloric acid (HCl), and then the corresponding PL spectra of N-L-CQDs solutions with different pH values at the excitation wavelength of 300 nm were detected.

The prepared N-L-CQDs aqueous solutions were maintained at different temperatures (−56, −20, 3, 25, and 50 °C) for 24 h. After that, the photoluminescence (PL) spectra of N-L-CQDs aqueous solutions were detected. Besides, the N-L-CQDs aqueous solutions were stored at room temperature, and the PL spectra were measured every few days. All of the PL spectra of N-L-CQDs were detected with the excitation wavelength of 300 nm.

#### 2.4.3. Cytotoxicity Assay

The cell viability of N-L-CQDs was measured by methyl thiazolyl tetrazolium (MTT) assay [33]. RAW264.7 cells (mouse macrophage) were incubated with Dulbecco’s modified medium (DMEM) supplemented with 10% fetal bovine serum (FBS). The RAW264.7 cells at a concentration of 1 × 10^4^ cells per mL were added to a 96-well plate in DMEM and cultured at 37 °C under 5% CO_2_ atmosphere. After 24 h, the cells were exposed to N-L-CQDs solutions with different concentrations (0, 2, 5, 10, 20, 50 μg/mL) to incubated for another 24 h. The cells were completely washed with phosphate buffered saline (PBS). After that, 200 μL MTT (0.5 mg/L) was added to each cell well and the cells were incubated for another 4 h. When the time of cell incubate expired, the culture medium was removed. Subsequently, 150 μL of dimethyl sulfoxide (DMSO) was seed to the wells in order to thoroughly dissolve the formazan crystals. The optical absorbance of each solution was measured at 570 nm by using a microplate reader (Bio-Rad, Hercules, CA, USA) to calculate the cell viability values, and the cell viabilities were estimated as the following formula.

$$\mathrm{Cell}\;\mathrm{viability}\;(\%)=(\frac{OD_{e}}{OD_{c}})\times 100\%$$

where *OD*_e_ is the absorbance of the experiment group and *OD*_c_ is the absorbance of the control group.

#### 2.4.4. Fluorescence Detection of Fe^3+^

The aqueous solution of FeCl_3_ (2 mL) with different concentration (0, 5, 10, 20, 50, 100, 200, 500, and 1000 μM) was added with a dispersive solution of N-L-CQDs (2 mL). The PL spectra ware performed at a maximum excitation wavelength of 300 nm after the mixture liquids equilibrated for 10 min.

#### 2.4.5. Selective Detection of Metal Cations

The N-L-CQDs mixed with different metal cations (Ca^2+^, Cd^2+^, Al^3+^, Mg^2+^, Mn^2+^, Pb^2+^, Zn^2+^, Ag^+^, Fe^3+^, Fe^2+^ Cu^2+^, Co^2+^, Cr^3+^, and K^+^) were detected to research the interference of metal cations. The as-prepared N-L-CQDs aqueous solution (2 mL) was mixed with different metal cations aqueous solution (2 mL, 0.2 mM), respectively. The fluorescence spectra were measured under excitation at 300 nm after retention for 10 min.

#### 2.4.6. Detection of Fe^3+^ in Real Water Samples

The experiment of fluorescence detection for Fe^3+^ was repeated by using river water in order to investigate the practicality of N-L-CQDs. The river water was obtained from Qinghe River in Beijing, China. The river water was used to prepare Fe^3+^ solutions with different concentrations (0, 5, 20, 50, 100, 200, 500, and 1000 μM).

## 3. Results and Discussion

### 3.1. Physiochemical Characterizations

**Figure 1:**
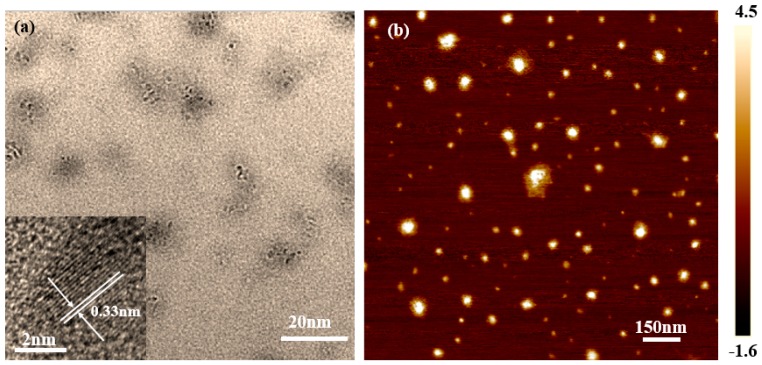
(**a**) TEM of N-L-CQDs. Inset: the HR-TEM of N-L-CQDs. (**b**) AFM photograph of N-L-CQDs.

The formation of N-L-CQDs was confirmed from TEM and AFM images as shown in Figure 1. The images of TEM and AFM clearly revealed that the as-prepared N-L-CQDs with a size distributed in a ranging from 4 to 12 nm were monodisperse and quasi-spherical. The inset of Figure 1a displayed the image recorded by high resolution TEM (HRTEM), indicating the average lattice spacing of 0.33 nm, which may be attributed to the (002) plane of graphite [44].

**Figure 2:**
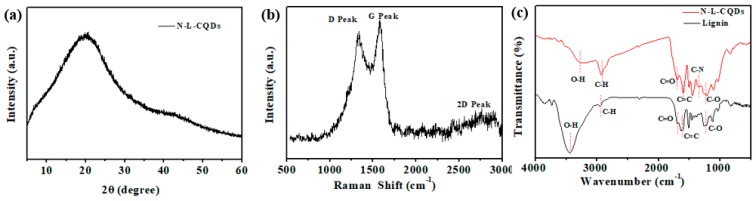
(**a**) XRD pattern of N-L-CQDs; (**b**) Raman spectrum of N-L-CQDs; and (**c**) FTIR spectra of N-L-CQDs.

The XRD pattern of N-L-CQDs was presented in Figure 2a and showed a distinct broad peak at 2θ = 20.96°, indicating the interlayer spacing of N-L-CQDs (0.42 nm) was broader than that of graphite (0.34 nm). This confirms the poor crystalline nature of carbon dots, which might be due to the oxygen containing groups existed on the surface of N-L-CQDs to enhance the interlayer distance [15]. Additionally, the Raman spectrum (Figure 2b) of N-L-CQDs clearly showed the characteristic D, G, and 2D bands. The D band at 1345 cm^−1^ was related to defect-induced structures of sp^3^ hybridized carbon atoms, while the G band at 1579 cm^−1^ could be assigned to the in-plane vibration of sp^2^ hybridized carbon atoms [45]. The 2D band at around 2750 cm^−1^ indicated the characteristics of few layers [31]. The relatively intensity of D band and G band (*I*_D_/*I*_G_) was estimated to be 0.934, which was similar to the graphite [43]. The FTIR spectra were recorded to further investigate the chemical structure of N-L-CQDs. The characteristic peaks of lignin observed (Figure 2c) at 3440 cm^−1^ (O–H stretching vibrations), 1697 cm^−1^ and 1230 cm^−1^ (stretching vibration of C=C and C–O). The characteristic absorption peaks of lignin could also be seen in the FTIR spectra of N-L-CQDs, which may be due to the relatively lower carbonization temperature and short standing time in the preparation of N-L-CQDs. Therefore, the N-L-CQDs were successfully prepared without destroying the core structure of lignin. However, comparing with the FTIR spectra of lignin, the O–H absorption of N-L-CQDs was obviously decreased, while C=O absorption at 1690 cm^−1^ was enhanced in N-L-CQDs. These results could be interpreted as esterification reaction between DES and lignin during the preparation of carbon quantum dots. Besides, the increasing of C=O absorption can also be assigned to the presence of carboxyl existed in DES. Meanwhile, the of C–H stretching vibrations at 2930 cm^−1^ enhanced and the C–N stretching vibrations emerged at 1346 cm^−1^ can be attributed to quaternary ammonium of DES. There results demonstrated that the successful doping of nitrogen in N-L-CQDs.

**Figure 3:**
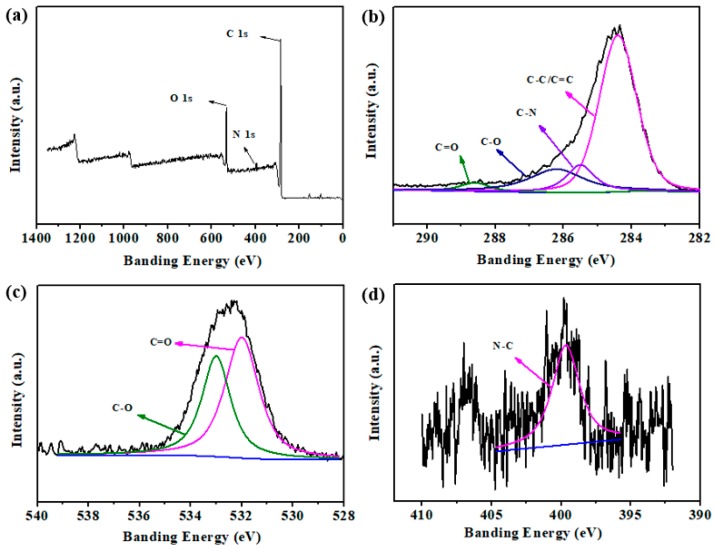
XPS wide scan spectrum of (**a**) N-L-CQDs; (**b**) high-resolution XPS spectra of C 1s; (**c**) O 1s; and (**d**) N 1s.

The XPS measurement was performed to further investigate the composition and content of atoms in N-L-CQDs. The survey spectra of N-L-CQDs can be seen in Figure 3. It can be observed that three predominant peaks at 533, 400, and 285 eV (Figure 3a), which could be assigned to the binding energies of O 1s, N 1s, and C 1s, respectively. The atomic ratio of the three elements in N-L-CQDs was 64.81% (C), 1.63% (N), and 33.56% (O), indicating the successful doping of nitrogen into carbon dots. The high-resolution spectrum of C 1s was shown in Figure 3b. The spectrum was divided into four peaks, including C–C/C=C bond with a binding energy at 284.4 eV; C–N bond at 285.5 eV; C–O bond at 286.2 eV; and C=O bond at 288.6 eV. Figure 3c shows the O 1s peak, which mainly consisted of two subpeaks at 532.07 eV and 533.18 eV, corresponding to C=O and C–O bands. As shown in Figure 3d, the N 1s spectrum indicated a transparent peak at 399.66 eV, which confirmed the dominant form of nitrogen as N–C group in N-L-CQDs. The data of XPS also showed the existence of plenty of hydrophilic function groups on the surface of N-L-CQDs. These results of XPS were consistent with that from FTIR spectra results.

### 3.2. Optical Properties of N-L-CQDs

**Figure 4:**
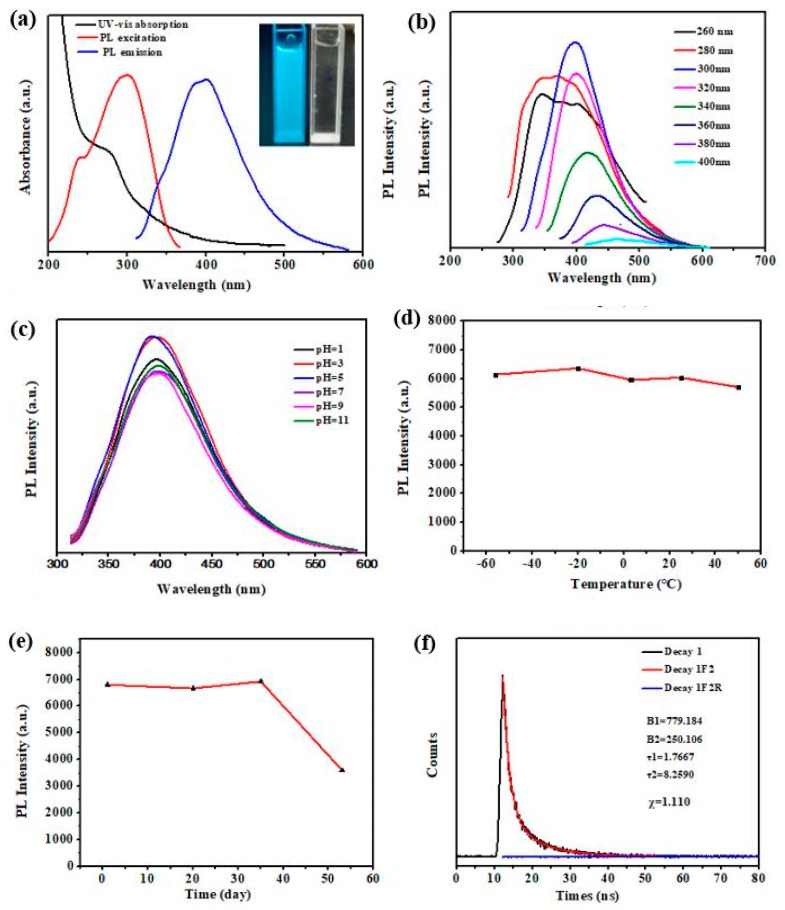
(**a**) UV–Vis absorption and fluorescence excitation and emission spectra of N-L-CQDs. Inset: the images of N-L-CQDs in aqueous solution under UV light of 365 nm (left) and sunlight (right). (**b**) PL emission spectra of N-L-CQDs excited by various wavelengths ranging from 260 nm to 400 nm with 20 nm increments. (**c**) Effect of pH values on PL intensity of N-LCQDs. (**d**) PL intensity change of N-L-CQDs solutions at different temperatures. (**e**) Effect of storage time on fluorescence intensity of N-L-CQDs solutions. (**f**) PL lifetime data of the N-L-CQDs.

CQDs usually exhibit strong absorption peak in the ultraviolet region (230–320 nm) and the tail extends into the visible range [46]. Figure 4a depicted the ultraviolet-visible (UV–Vis) absorption and photoluminescence (PL) spectra for the N-L-CQDs. The UV–Vis absorption spectrum of N-L-CQDs showed a broad absorption shoulder at around 280 nm, which typically ascribed to the π–π* transition of aromatic C=C bonds and the n–π* transition of C=O bonds [31,47]. It can be clearly seen from the PL spectra that the maximum emission peak of N-L-CQDs appeared around 400 nm upon excitation wavelength at 300 nm. The inset photograph of the N-L-CQDs aqueous solution was nearly transparent under daylight and showed bright blue fluorescence under the irradiation of ultraviolet light (Figure 4a).

The PL spectra of N-L-CQDs were assessed to further investigate the optical properties of N-L-CQDs. The fluorescence spectra of the N-L-CQDs at various excitation wavelengths can be observed in Figure 4b. The emission peak was shifted from 380 to 466 nm with increasing excitation wavelength from 260 to 400 nm. When PL spectra of N-L-CQDs were excited from 260 to 300 nm, the emission peaks were gradually red-shifted with increased of intensity. The strongest emission peaks were gradually decreased and continued red shift as a result of the shifting of PL spectra excited from 300 to 400 nm. The emission spectra showed an excitation-dependent feature, which can be attributed to the function of the oxygen and/or nitrogen functional groups of N-L-CQDs [31]. What is more, the phenomenon of the excitation-dependent and red-shift was also similar to previous reports [48,49]. The PL intensity stability of N-L-CQDs with different pH values ranging from 1 to 11 was also investigated (Figure 4c). The PL emission wavelengths of N-L-CQDs remained relatively stable in the pH range from 1 to 11. The PL intensity was relatively stable in alkaline condition. Under the acidic condition, the fluorescence intensity was greatly increased. However, the PL intensity had a certainly decline in strong acidic condition. The fluorescence stability of N-L-CQDs aqueous solutions for 24h preservation under different temperatures (−56, −20, 3, 25, and 50 °C) was further investigated. As shown in Figure 4d, it is worth noting that the fluorescence intensity of N-L-CQDs aqueous solutions kept almost unchanged as treated at different temperatures ranging from −56 to 50 °C. Besides, the effect of storage time on fluorescence intensity of N-L-CQDs solutions was analyzed, as shown in Figure 4e. The PL intensity of N-L-CQDs aqueous solutions remained relatively stable at room temperature within 35 days, whereas it dramatically became unstable after 35 days. These results indicated that the N-L-CQDs possessed excellent fluorescence stability, which would be beneficial to expand the range of applications for N-L-CQDs.

The PL decay and the biexponential fitting curves of N-L-CQDs can be seen in Figure 4f. The decay curve was fitted as follows

Y_(t)_ = *A*_1_exp(−*t*/τ_1_) + *A*_2_exp(−*t*/τ_2_)

where *A*_1_ and *A*_2_ are the fractional intensities, *t* is time, and τ_1_ and τ_2_ are decay lifetime. The biexponential behavior of lifetime indicates that there are two different emission sites, which means that the state of fluorescence was caused by the center of graphite (or conjugated structure) and surface traps [50]. The parameters obtained by using instrument response function (IRF) and can be observed in inset of Figure 4f. The average fluorescence lifetime was calculated by the following formula

τ_ave_ = (*A*_1_τ_1_^2^ + *A*_2_τ_2_^2^)/(*A*_1_τ_1_ + *A*_2_τ_2_)

The average fluorescence lifetime (τ_ave_) of N-LCQDs was calculated as 5.68 ns (χ < 1.10). Furthermore, the fluorescence quantum yield (QY) of N-L-CQDs was generated to be 7.95%, which was comparable to other reported results [50].

### 3.3. Detection of Fe^3+^ Ions

**Figure 5:**
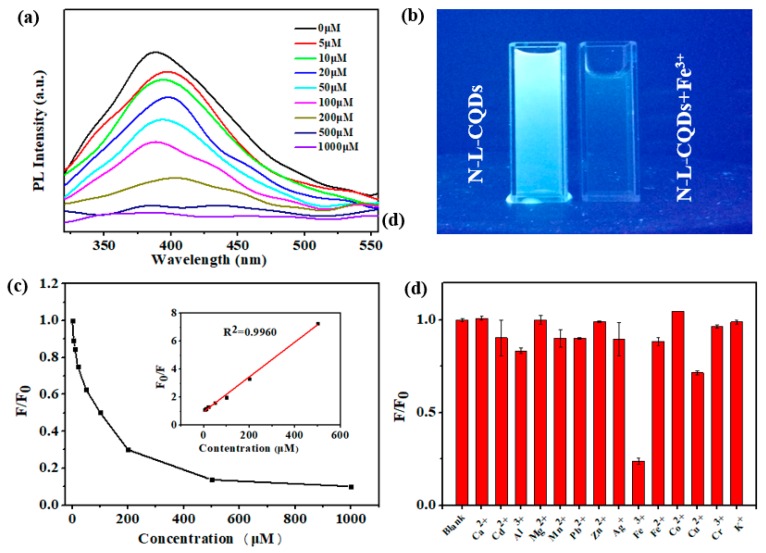
(**a**) Fluorescent emission spectra of N-L-CQDs quenched by Fe^3+^ with various concentrations (from top to bottom: 0, 5, 10, 20, 50, 100, 200, 500, and 1000 μM). (**b**) The photographs of N-L-CQDs aqueous solutions without (left) and with Fe^3+^ under ultraviolet light (365 nm). (**c**) Relative PL intensity of N-L-CQDs in the presence of increasing concentration of Fe^3+^. Insert: a linear relationship of *F*_0_/*F* on the concentration of Fe^3+^ ranging from 0 to 500 μM. (**d**) Selective PL response of N-L-CQDs solution after added different metal ions with concentration of 200 μM.

Based on the strong photoluminescence of N-L-CQDs and high affinity between Fe^3+^ ions and N-L-CQDs, the Fe^3+^ ions with different concentrations (from 0 to 1000 μM) were add to the N-L-CQDs solution to detect the sensitivity of N-L-CQDs for Fe^3+^ and the results were shown in Figure 5a. The PL intensity of the N-L-CQDs at 300 nm was gradually decreased in the presence of Fe^3+^ range from 0 to 1000 μM, which revealed that Fe^3+^ can be detected by the N-L-CQDs. The fluorescence changes of N-L-CQDs in the presence and absence of Fe^3+^ can be observed by naked eye in ultraviolet lamp (365 nm) and the photography was shown in Figure 5b. The aqueous solution of N-L-CQDs exhibited strong blue fluorescence (left) under UV lamp (365 nm), while it displayed faint blue in the presence of Fe^3+^ ions (right).

The changing ratio of relative fluorescence intensity (*F*/*F*_0_) with the increasing of Fe^3+^ was described in Figure 5c, in which *F* and *F*_0_ were the PL intensities of N-L-CQDs solutions in the presence and absence of Fe^3+^ ions. The inset of Figure 5c exhibited a good linear relationship between the relative fluorescence intensity (*F*_0_/*F*) and concentration of Fe^3+^ from 0 to 500 μM. The linear relationship can be analyzed by the Stern–Volmer equation, and the equation was presented as follows

$$\frac{F_{0}}{F}=1+K_{SV}C$$

*K*_SV_ signifies the quenching constant and *C* is the concentration of Fe^3+^. The *K*_SV_ of linear regression equation is 1.24 × 10^−2^ with a coefficient of determination (*R*^2^ = 0.9960). The detection limit for Fe^3+^ was calculated to be 0.44 μM, which was comparable to the mostly reported quantum dots. For example, Xie et al. synthesized CQDs as fluorescent probes for detection of iron ion with a detection limit of 13.68 μM [51]. Moreover, ZnTe quantum dots was also prepared for the detection of Fe^3+^ with a detection limit of 4.89 μM [52]. However, there are still some groups have successfully synthesized CQDs with superior sensitivity for Fe^3+^ than N-L-CQDs. Deng et al. have prepared N-CQDs using biomass tar as the carbon precursor for the detection of Fe^3+^ with a detection of 60 nM [53]. In this study, the detection limit of N-L-CQDs for Fe^3+^ (0.44 μM) was far below the maximum tolerance (5.4 μM) of Fe^3+^ in drinking water [29]. There results suggested that the N-L-CQDs exhibited superior sensitivity response to Fe^3+^ and could be promising for applying in the detection of Fe^3+^.

We also utilized the N-L-CQDs as probes to detect other metal ions (200 μM), including Ca^2+^, Cd^2+^, Al^3+^, Mg^2+^, Mn^2+^, Pb^2+^, Zn^2+^, Ag^1+^, Fe^3+^, Fe^2+^ Cu^2+^, Co^2+^, Cr^3+^, and K^+^. Then selective PL response of N-L-CQDs for different metal ions ware recorded, and the results were shown in Figure 5d. Obvious fluorescence quenching of the solution of N-L-CQDs was generated by adding of Fe^3+^, while other metal ions had no great effect on fluorescent intensity of N-L-CQDs. These results indicated that fluorescent sensor of N-L-CQDs was highly selective for Fe^3+^ and can be used as fluorescent probes for the detection of Fe^3+^ in aqueous solution. The reason for fluorescence quenching could be attributed to the formation of N-L-CQDs-Fe^3+^ complexes, in which the electronegativity O and N groups (such as phenolic hydroxyl, carboxyl, amino groups, etc.) on the surface of N-L-CQDs can associate with metal ions to form coordination complexes [53,54,55]. Once the N-L-CQDs-Fe^3+^ complexes were formed, the electrons in the excited state of N-L-CQDs surface were easily transferred to half-filled 3d orbitals of Fe^3 +^ and accompanied by energy transfer forming non-radiative recombination. In addition, the high selectivity of N-L-CQDs for Fe^3 +^ might also be assigned to the higher thermodynamic affinity of Fe^3 +^ than other metal ions and quicker chelating with N-L-CQDs [29].

**Figure 6:**
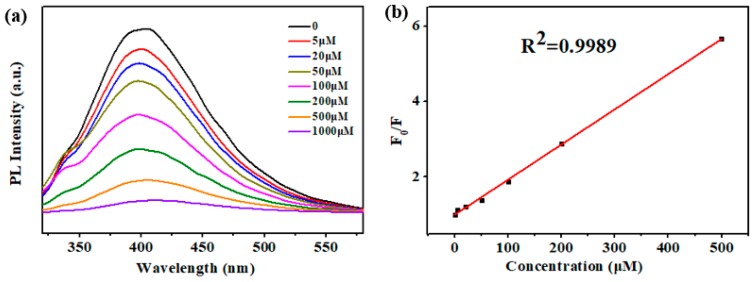
(**a**) Fluorescent emission spectra of N-L-CQDs quenched by Fe^3+^ with various concentrations (from top to bottom: 0, 5, 20, 50, 100, 200, 500, and 1000 μM) in river water. (**b**) A linear relationship of *F*_0_/*F* on the concentration of Fe^3+^ ranging from 0 to 500 μM).

Fe^3+^ ions with different concentrations (from 0 to 1000 μM) in river water were add to the N-L-CQDs solutions in order to analyze the practicality of N-L-CQDs. The fluorescent spectra of N-L-CQDs with different concentrations of Fe^3+^ ions are shown in Figure 6a. It was noted that PL intensity gradually decreased when the concentration of Fe^3+^ ions was increased from 0 to 1000 μM. More importantly, when concentrations of Fe^3+^ ions was 5 μM, the PL intensity was decreased obviously. All the results were similar to the experiment of fluorescence detection for Fe^3+^ in deionized water as shown in Figure 5a. However, it was shown that the obvious PL intensity was still observed when Fe^3+^ ions concentration was 500 μM in river water, while it disappeared in the deionized water. Besides, the relative fluorescence intensity (F_0_/F) and concentration of Fe^3+^ from 0 to 500 μM also exhibited a good linear relationship (Figure 6b). Although the detection of Fe^3+^ ions might be interfered by some impurities, the N-L-CQDs still exhibited excellent sensitivity for Fe^3+^. Therefore, the N-L-CQDs can be applied to detect Fe^3+^ in practical environmental detection.

### 3.4. Cytotoxicity Evaluation

**Figure 7:**
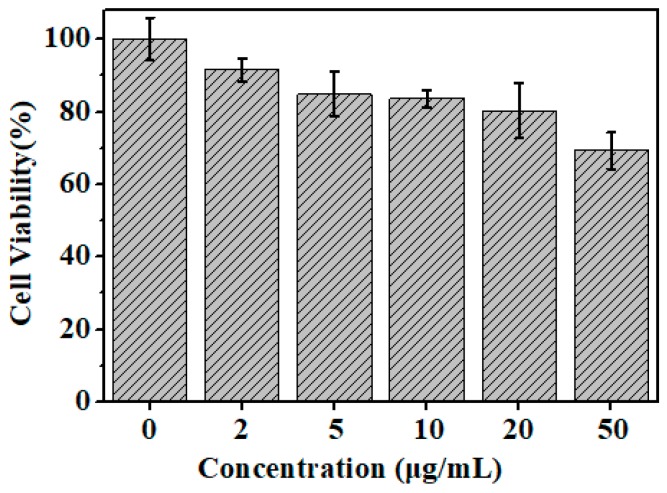
Cytotoxicity of N-L-CQD against mouse macrophage cells at different concentrations from 0 to 50 μg/mL.

The remarkable feature of CQDs is their small particle size, excellent optoelectronic properties, which suggested that the CQDs are suitable for biomedical applications, such as drug delivery and cells imaging. Therefore, it is necessary to measure the compatibility between cells and CQDs. The cell viability of N-L-CQDs was evaluated by MTT assay using macrophage cells. The result of cell viability was obtained after the cells incubated in N-L-CQD solution at different concentrations from 0 to 50 μg/mL for 24 h as shown in Figure 7. The results revealed that the cell viability was more than 80% after 24 h incubation in N-L-CQDs at concentration of 20 μg/mL. The viability of cells was over than 70% when the concentration of N-L-CQDs was reached to 50 μg/mL. These results suggested that N-L-CQDs did not exert potential toxicity at a concentration of 20 μg/mL. Therefore, the concentration of N-L-CQDs at 20 μg/mL can be used for biomedical applications.

## 4. Conclusions

In this work, nitrogen-doped lignin carbon quantum dots (N-L-CQDs) were successfully prepared with an easy and environmentally friendly approach. Alkali lignin was used as a cheap and readily available main carbon source. For the first time, DES was used as a carbon source, nitrogen source, and a solvent of alkali lignin. The as-prepared N-L-CQDs with a quantum yield of 7.95% exhibited strong and stable bright luminescence. The N-L-CQDs demonstrated excellent sensitivity and selectivity by quenching to Fe^3+^ ions with detection limit of 0.44 μM, indicating that N-L-CQDs can used as a fluorescent probe for the detection of ferric ions. Besides, they may also be used in biomedical due to the excellent fluorescence and low cytotoxicity of N-L-CQDs at a concentration of 20 μg/mL.
